# Research to improve the health of vulnerable populations

**DOI:** 10.2471/BLT.17.030217

**Published:** 2017-02-01

## Abstract

Mani Jegathesan tells Fiona Fleck how his carriers in tropical disease research and athletics combine to serve public health.

Q: How did you become interested in medicine and public health?

A: When I started school in Malaysia it was a British colony. But by the time I went to university, it was an independent country. I was one of those transitional people also in the sense that my father was an immigrant from Sri Lanka. In the migrant community, our parents extolled us to become a doctor, a lawyer or an engineer. The first one made most sense for me (at that time).

Q: How did you become interested in sports?

A: My father had a natural talent for running but could not participate in sports events as they were reserved for the British community. That changed in 1924. Given a chance to compete nationally, he promptly went on to win one of these events. After independence in 1957, he became a sports organizer and set up Malaysia’s Olympic Council and other national sports associations. My elder brother competed in the Melbourne Olympics in 1956. It was quite inspirational and – perhaps something to do with the DNA – so it was not unnatural for me to follow suit. My family gave me a great deal of encouragement and by the time I left secondary school in Singapore, I was the fastest runner in Malaysia and Singapore.

Q: You won three gold medals at the Asian Games in Bangkok in 1966, including the title of “fastest man in Asia”, and became the first Malaysian to enter the semi-final stage of an Olympic event, the 200 metres, in 1964, repeating this in 1968 and setting a national record which stands unbroken today. How did your sporting successes feed into your efforts to improve public health in your country?

A: Two things. One, I realized that even in elite sports you are not necessarily an elitist, as you can motivate grassroots sports and physical activities in clubs and schools. I realized from very early on the benefits of physical activity as one of the cornerstones of a healthy life and something that can keep a whole community healthy. Two, perhaps sports gave me the opportunity to work at the national Institute for Medical Research, when I was invited by a leading scientist who happened to be a sports fan. One day he saw me having lunch and said, “Let’s get this guy in our institute”. I joined the institute a few days later and stayed there for 32 years, including as its director.

“People were still dying of diarrhoeal diseases, such as cholera and typhoid.”

Q: You were among the pioneers in research on tropical diseases in your country. What were the challenges when you started out in the field?

A: In the 1960s and 1970s, people were still dying of diarrhoeal diseases, such as cholera and typhoid; acute respiratory infections, such as pneumococcus pneumonia, parasitic infestations such as malaria and there were heavy disease burdens from filariasis and helminthiasis. During the course of my career in research and public health, infectious diseases declined in our country. The standard of living improved, including improvements in the provision of clean water and sanitation, and the health ministry was quite successful in creating an accessible and efficient health system and in overseeing the management and treatment of infectious disease cases. As a result I witnessed a shift from infectious to chronic diseases, such as heart disease, stroke and diabetes. 

Q: What led to your interest in research and its application in public health? 

A: As a young trainee I was sent out to the field. At the time, there was a great belief in the vaccine gun. I would take one with me and immunize hundreds of people against cholera every day. It was a major ritual. People lined up in schools and markets and thanks to a combination of other health as well as developmental measures we saw the removal of cholera as a menace. There are still a few cholera cases in Malaysia, but people do not die of this anymore. I saw the problems and participated in the solutions. The World Health Organization (WHO) recognized our value early on and established a network of research centres. Our institute was selected as one of those collaborating centres for research on tropical diseases in the Western Pacific region.

Q: How did your research address neglected tropical diseases and health conditions affecting the poorest of the poor, the most vulnerable populations?

A: At the Institute for Medical Research – on its own and through its regional affiliations with WHO and SEAMEO TROPMED – we had insight into the health problems confronting countries in our region. Based on this, we developed a research agenda and projects focused on tropical diseases with a great public health burden. Most importantly, we had dedicated researchers and we worked as teams. We knew that these diseases affected mostly rural folk, who were often economically disadvantaged. Treating these diseases was a major imperative for our nation to advance towards developed country status. 

Q: What kind of research did you do? 

A: I did research on emerging bacterial infections in Malaysia, such as Pseudomonas* pseudomallei,* new serotypes and biotypes of *salmonellae* and on antimicrobial resistance, including the first cases in our country of penicillin-resistant *gonococci*, methicillin-resistant *staphylococci* and choloramphenicol-resistant Salmonella* typhi*. Our research findings were later used to inform health ministry guidelines on infection control and the use of disinfectants in hospitals. Our findings also led to a greater use of laboratory-based epidemiological markers to detect salmonella, shigella and other bacteria to control outbreaks.

Q: A key development that also gave impetus to research for infectious diseases in low- and middle-income countries was the establishment in 1973 of the Joint Coordinating Board of the Special Programme for Research and Training in Tropical Disease (TDR). You were invited to be the rapporteur at some of the first meetings, can you tell us about that?

A: The Joint Coordinating Board was established to foster, support and fund the activities of TDR as well as to ensure good governance for the programme. At that time the priorities were parasitic diseases, malaria, filariasis, leishmaniasis and the like. Also, diarrhoeal diseases and acute respiratory infections were responsible for high mortality especially among children in the least developed countries and these were rightly allocated their own programmes at WHO. I also participated in meetings associated with these programmes during my tenure at the Ministry of Health in Malaysia.

Q: The Council for Health Research for Development (COHRED) was set up to promote research in countries with a high burden of infectious diseases. Can you tell us about your work with this organization?

A: The focus of our research institute in Malaysia was mainly epidemiological, biomedical and laboratory diagnosis, and that was fairly typical for many research institutes across south-east Asia. In the 1980s, we recognized that behavioural factors and therefore social science and research were as important as biomedical sciences for addressing public health challenges. This trend was influenced by WHO in Geneva and was part of a movement that promoted the concept of essential national health research. 

Q: What is essential national health research? 

A: The idea was to empower developing countries to conduct the research that was relevant to their health needs. That meant research that defined and measured their health problems and that proposed solutions, along with monitoring and evaluating measures to ensure that those solutions were right. COHRED encouraged low- and middle-income countries to participate in their network so that we could all work towards this objective. I started working with COHRED to promote this new type of collaboration and research in countries. I worked closely with researchers in Nepal, Pakistan, South Africa, Thailand, [the United Republic of] Tanzania and Zimbabwe, and in many other countries.

Q: Collaborations became increasingly important for research in developing countries. How were you involved? 

A: I worked with SEAMEO TROPMED, a network through which south-east Asian countries could collaborate on common health issues by building knowledge and research capacity to tackle endemic infectious diseases. We did this by sharing resources, both infrastructure and personnel. For example, higher education courses were assigned to specific countries. Thailand took on the Diploma in Tropical Medicine course; Malaysia, the Diploma in Applied Parasitology and Entomology while the Philippines took on Public Health. This education collaboration expanded to more countries and courses, giving young scientists from across the region the chance to train in different disciplines, and to enable research projects to be formulated and carried out in the field in these countries. It meant we could take research to the places where these diseases affected people the most, often the most disadvantaged and economically challenged populations in the countries concerned.

“We could take research to the places where these diseases affected people the most, often the most disadvantaged.”

Q: How successful has your country been in addressing these and other public health challenges? 

A: We have witnessed a dramatic improvement in life expectancy, infant mortality and the reduction of infectious diseases in our country and in Asia as a whole. There are still major challenges, such as reducing malaria and in controlling other mosquito-borne diseases such as dengue, chikungunya and Zika. Today, however, the main challenge is the rising prevalence of chronic diseases. In recent years, I have been involved in the health ministry’s Healthy Lifestyle campaign and a campaign, called Active Malaysia, led by the Ministry of Youth and Sports to make people more aware of the need for exercise and its health benefits. As a motivational speaker and adviser, I talk to government agencies, professional groups and the public about leading a healthy lifestyle as one of the best forms of prevention of disease and the answer to many of today’s health issues.

